# Amino-Functionalized Lead Phthalocyanine-Modified Benzoxazine Resin: Curing Kinetics, Thermal, and Mechanical Properties

**DOI:** 10.3390/polym11111855

**Published:** 2019-11-11

**Authors:** Li-wu Zu, Bao-chang Gao, Zhong-cheng Pan, Jun Wang, Abdul Qadeer Dayo, Wen-bin Liu

**Affiliations:** 1 Institute of Composite Materials, College of Materials Science and Chemical Engineering, Harbin Engineering University, Harbin 150001, China; zlwsm@163.com (L.-w.Z.); chemgbc@hrbeu.edu.cn (B.-c.G.); 2 College of Materials Science and Engineering, Heilongjiang Province Key Laboratory of Polymeric Composition, Qiqihar University, Qiqihar 161006, China; 3 Department of Chemical Engineering, Engineering and Management Sciences, Balochistan University of Information Technology, Quetta 87300, Pakistan

**Keywords:** benzoxazine resin, phthalocyanine, curing kinetics, thermosetting resin

## Abstract

Phenol-diaminodiphenylmethane-based benzoxazine (P-ddm)/phthalocyanine copolymer was prepared by using P-ddm resin as matrix and 3,10,17,24-tetra-aminoethoxy lead phthalocyanine (APbPc) as additive. Fourier-transform infrared (FTIR), differential scanning calorimetry (DSC), dynamic mechanical analysis (DMA), and thermogravimetric analysis (TGA) were used to investigate the curing behavior, curing kinetics, dynamic mechanical properties, thermal stability, and impact strength of the prepared copolymers. The kinetic parameters for the P-ddm/APbPc blend curing processes were examined by utilizing the iso-conversional, Flynn–Wall–Ozawa, and Málek methods. The P-ddm/APbPc blends exhibit two typical curing processes, and DSC results confirmed that the blending of APbPc monomer can effectively reduce the curing temperature of P-ddm resin. The autocatalytic models also described the non-isothermal curing reaction rate well, and the appropriate kinetic parameters of the curing process were obtained. The DMA and impact strength experiments proved that the blending of APbPc monomer can significantly improve the toughness and stiffness of P-ddm resin, the highest enhancements were observed on 25 wt.% addition of APbPc, the recorded values for the storage modulus and impact strength were 1003 MPa and 3.60 kJ/m^2^ higher, respectively, while a decline of 24.6 °C was observed in the glass transition temperature values. TGA curves indicated that the cured copolymers also exhibit excellent thermal stabilities.

## 1. Introduction

Thermosetting resins, such as phenolic resins, epoxides, bismaleimides, cyanates, vinyl esters, and polyimides, are high-performance polymers with a wide range of applications in aerospace, structural, adhesives, electronics, and coatings fields. In recent years, polybenzoxazine resins have gained immense attention in academia and industry, because they combine the thermal and mechanical strength of phenolic resins with much wider molecular design flexibility and overcome several disadvantages of traditional phenolic resins [1,2,3,4,5,6,7,8,9,10,11,12]. These resins have a number of excellent properties such as high carbon yield, low water absorption, high mechanical strength, high glass transition temperature (*T_g_*), very low shrinkage, and no byproduct formation during the curing process. However, polybenzoxazine still has shortcomings such as being cured at higher temperature, processing difficulty, and higher brittleness [7,8,9,13]. Commonly two methodologies are employed to overcome these limitations, (i) altering the benzoxazine monomer structure by molecular design, as new functionalities can be introduced by modifying the structure of the main chain [14,15,16,17], side chain [18,19,20], or end chain structure [21,22,23,24], (ii) by adding a second component such as nano-inorganic particles [25,26,27,28], rubber elastomers [29], fiber [30], or thermoplastic polymers [31,32,33].

The phthalocyanine (Pc) compound is a kind of organic dye with extraordinary physicochemical properties, photochemical stability, and complex aggregation behavior. Pc constitutes a group of heterocyclic compounds with four isoindole-class [(C_6_H_4_)C_2_N] units linked by four N atoms to form a conjugated macrocyclic chain, a number of Pc are modified in the center with various metal elements, including Cu, Zn, Pb, etc., and substituted in the periphery with various groups [34,35,36,37]. Metal phthalocyanine has been widely used in optical, conducting, and magnetic materials; dye-sensitized solar cells; organic thin-film transistors; organic light-emitting devices; fuel cells; catalysis; sensors; and photodynamic therapy [38,39,40]. Pb and Pb-containing inorganic or organic compounds are most commonly used as high-energy ray radiation protection materials because of their low cost and excellent properties of radiation shielding. Pb and Pb-containing polymer matrix composites with light-weight shielding materials have been successfully used in medical settings for technician and patient protection in the field of X-ray, equipment containers, and applications of nuclear energy [41,42,43]. However, inorganic compounds such as PbO and PbO_2_ are difficult to evenly disperse in the polymer matrix due to their high density. Thus, lead phthalocyanine with aliphatic amine groups may become a potential candidate in polymer matrix radiation shielding materials.

In the current study, amino-functionalized lead phthalocyanine (APbPc) is blended in phenol-diaminodiphenylmethane-based benzoxazine (P-ddm) in different wt.% from 5% to 25%. The curing kinetics of P-ddm/APbPc blends, the dynamic mechanical properties, toughening mechanism, and thermal stabilities of P-ddm/APbPc copolymer were studied to understand the effects of the APbPc blending on the P-ddm benzoxazine. With the introduction of amine groups containing lead phthalocyanine, the curing temperature of benzoxazine monomer decreased. Furthermore, amine groups can crosslink with the opening oxazine ring to form homogeneous copolymers. APbPc can better disperse in the polybenzoxazine network and maintain the structural stability of benzoxazine resin under exposure to high-energy radiation.

## 2. Materials and Methods

### 2.1. Materials

The P-ddm monomer (99.5%) was kindly donated by Jiangxi Huacui Advanced Materials Co., Ltd., Jiangxi, China and 3,10,17,24-tetraaminoethoxy lead phthalocyanine (APbPc) was synthesized in the laboratory according to literature [44,45]. FTIR and ^1^H spectra of the APbPc monomer were conducted for the purity analysis (Figure S1).

### 2.2. Methods

#### 2.2.1. Preparation of P-ddm/APbPc Copolymers

An appropriate mass of APbPc monomer as per 5, 10, 15, 20, and 25 wt.% of the total mass was added into P-ddm resin at 90–100 °C. The blend was vigorously stirred for next 30 min and transferred into steel molds having test dimensions and vacuumed for 30 min. Then the blends were moved to a heating oven and cured by the following curing process—150 °C for 2 h, 170 °C for 4 h, and 190 °C for 1 h. The copolymer samples were removed from the room temperature cold mold and polished with sand paper to meet the testing dimensions.

#### 2.2.2. Characterizations and Measurements

The FTIR spectra were recorded on a Bruker Vertex 70 Instruments (Bruker Austria GmbH, Vienna, Austria) equipped with a golden gate single reflection ATR accessory, in 4000–500 cm^−1^ spectrum range. Differential scanning calorimetry (DSC) analysis was done using Q200 (TA Instruments, New Castle, PA, USA), with α-Al_2_O_3_ as reference, at a heating rate of 20 °C/min in nitrogen, (flow rate is 30 mL/min). The scanning temperature range was from 20 to 300 °C. Thermogravimetric analysis (TGA) was performed using Q50 (TA Instruments, New Castle, PA, USA) at a 10 °C/min heating rate from 25 to 800 °C. The initial mass of the samples was 6–10 mg. The dynamic mechanical analysis (DMA) was performed from 25 to 275 °C on a Q-800 (TA Instruments, New Castle, PA, USA). The sample size was 30 mm × 5 mm × 2 mm. The single-cantilever mode was adopted with 3 °C/min heating rate, 1 Hz frequency, and 10 microns amplitude.

The crosslink density of the P-ddm/APbPc copolymers was calculated from the equilibrium storage modulus in the rubbery region. This equation better described the elastic properties of polybenzoxazines. The equation is represented as (1) [46,47]:

(1) $$\rho =G^{\prime }/3\phi \;RT$$

where *G′* is the equilibrium elastic modulus in the rubbery plateau, *ϕ* is a front factor, which is unity for ideal rubbers, *R* is the ideal gas constant (8.314 J/mol·K), *T* is the absolute temperature, and *ρ* (mol/m^3^) is the crosslink density, which is the mole number of network chains per unit volume of the polymers.

Non-isothermal DSC was used to study the curing kinetics. In curing kinetics analysis, it is basically assumed that the exothermic heat (*dH/dt*) is proportional to the reaction conversion rate, (*dα/dt*). The reaction-conversion rate at a given time is considered to be the function of cure degree, *α*. Based on the above assumptions and the Arrhenius equation, a series of dynamic analysis formulae can be obtained as follows [46,48,49,50]:

(2) $$\frac{d\alpha }{dt}=\frac{dH/dt}{\Delta H_{0}}=\beta \frac{d\alpha }{dT}=K\left(T\right)f\left(\alpha \right)$$

(3) $$\alpha =\frac{\Delta H_{i}}{\Delta H_{0}}$$

(4) $$\frac{d\alpha }{dt}=\beta \frac{d\alpha }{dT}=Aexp\left(\frac{-E_{a}}{RT}\right)f\left(\alpha \right)$$

where (*dH/dt*) is the heat flow, $\Delta H_{i}$ is the total exothermic enthalpy of the reaction from 0 to time *t*, $\Delta H_{0}$ is the total exothermic enthalpy of curing reaction. *A* is the pre-exponential factor. *E_a_* is the activation energy (kJ/mol), β is the heating rate (K/min), *R* is the ideal gas constant (8.314 J/mol·K), *T* is the absolute temperature.

Model-free isoconversional methods can be used to study how the activation energy changes throughout the entire reaction. Isoconversional methods can give accurate values of the apparent activation energy *E_a_*, the Flynn–Wall–Ozawa (FWO) is one of the most used models and is represented by the following equations [51,52]:

(5) $$\ln\beta =\ln\frac{AE_{a}}{R}-\mathrm{lng}\left(\alpha \right)+5.331-1.052\frac{E_{a}}{RT}$$

(6) $$g\left(\alpha \right)=\int_{0}^{\alpha }\frac{d\alpha }{f\left(\alpha \right)}$$

where g(α) is the integral conversion function.

Isoconversional methods are very suitable for accurate estimation of the *E_a_*, but they do not assume any reaction model. According to different cure mechanisms, the cure kinetic model can be divided into n-order model and autocatalytic (Šesták–Berggren) model [53,54]. These models are expressed as Equations (7) and (8), respectively, where m and n are the reaction orders:

(7) $$\frac{d\alpha }{dt}=\beta \frac{d\alpha }{dT}=Aexp\left(\frac{-E_{a}}{RT}\right){\left(1-\alpha \right)}^{n}$$

(8) $$\frac{d\alpha }{dt}=\beta \frac{d\alpha }{dT}=Aexp\left(\frac{-E_{a}}{RT}\right)\alpha^{m}{\left(1-\alpha \right)}^{n}$$

In order to determine the kinetic triplet (*E_a_*, *A*, and *f*(*α*)), it is necessary to ascertain the reaction model. One of the most useful methods is the use of the Málek method, According to the Málek method, two special functions *y*(*α*) and *z*(*α*) can be used to determine a kinetic model and then estimate the kinetic parameters by using Equations (9) and (10), with *u = Ea/RT*. π(u) is shown in Equation (11). According to the Senum and Yang fourth-order rational equation, the value *π*(*x*) can be approximately calculated [46,48].

(9) $$y\left(\alpha \right)=\frac{d\alpha }{\mathrm{dt}}\cdot e^{u}$$

(10) $$z\left(\alpha \right)=\pi \left(u\right)\cdot \frac{d\alpha }{\mathrm{dt}}\cdot \frac{T}{\beta }$$

(11) $$\pi \left(u\right)=\frac{u^{3}+18u^{2}+88u+96}{u^{4}+20u^{3}+120u^{2}+240u+120}$$

## 3. Results and Discussion

### 3.1. FTIR Analysis

**Figure 1:**
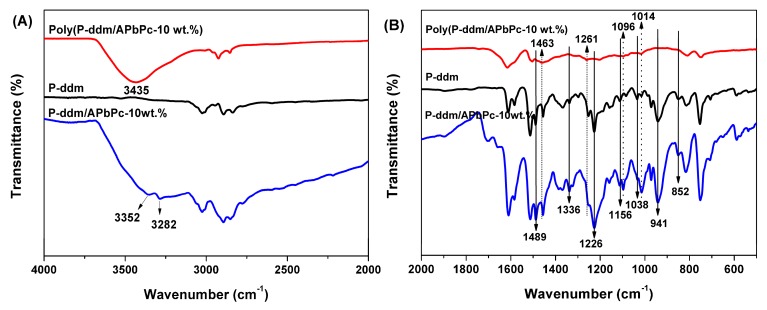
FTIR spectra of neat phenol-diaminodiphenylmethane-based benzoxazine (P-ddm) resin, P-ddm/APbPc (3,10,17,24-tetra-aminoethoxy lead phthalocyanine) blend, and P-ddm/APbPc copolymer. (**A**) 2000–4000 cm^−1^, (**B**) 600–2000 cm^−1^.

**Scheme 1:**
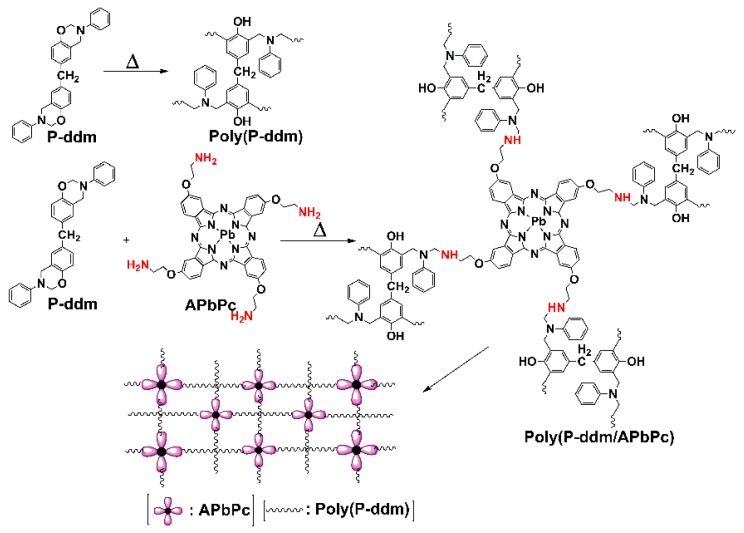
Proposed crosslinked network structure for P-ddm/APbPc copolymer.

Figure 1 shows the FTIR spectra of neat P-ddm monomer, P-ddm/APbPc blend, and P-ddm/APbPc copolymer. The benzoxazine characteristic absorption bands at 1489 (*ortho*-disubstituted benzene), 1336 (CH_2_ wagging), 1226 (asymmetric stretching of C–O–C), 1156 (asymmetric stretching of C–N–C), and 1038 cm^−1^ (symmetric stretching of C–O–C) was observed in the neat P-ddm resins and P-ddm/APbPc blend. The bending vibration of C–H located at 941 cm^−1^ was due to the characteristic mode of benzene with an attached oxazine ring [24,34,55]. For P-ddm/APbPc, we could also see the stretching vibrations of N–H at 3352 and 3282 cm^−1^ and skeleton vibrations of the phthalocyanine ring at 1096 and 1014 cm^−1^ besides the characteristic modes of oxazine ring [56]. After curing, all major characteristic absorption peaks of oxazine ring and NH_2_ groups of APbPc for poly(P-ddm/APbPc) completely disappeared. The new peaks at 3435, 1463, and 1261 cm^−1^ were assigned to phenolic hydroxyl groups produced from oxazine ring-opening, tetrasubstituted benzene ring mode, and C–N linked between an amino group of APbPc and poly(P-ddm), respectively [41]. This suggested that Mannich bridge linkage and phenolic groups are formed [30,57], and amino groups of the APbPc take part in the reaction. Based on the FTIR observation we proposed the networked structure for P-ddm/APbPc in Scheme 1, we conclude poly(P-ddm/APbPc) has formed a crosslinked structure that will possibly improve the mechanical and thermal properties as described in the following sections.

### 3.2. Curing Behavior of P-ddm/APbPc Blends

**Figure 2:**
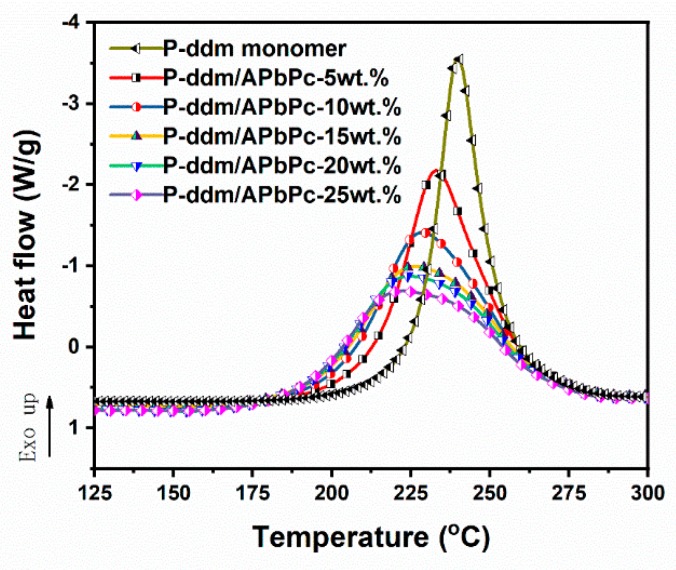
Differential scanning calorimetry (DSC) curves of P-ddm/APbPc blends at different wt.% of APbPc.

Figure 2 shows the DSC spectra of the P-ddm/APbPc blending system with different wt.% of APbPc, and the characteristic curing temperature and exothermic enthalpy of the system are summarized in Table 1.

The initial curing temperature (*T_o_*) and peak curing temperature (*T_p_*) were decreased from 227.2 and 241.0 °C to 185.6 and 222.5 °C, respectively, reducing by 41.6 and 18.5 °C on the addition of 25 wt.% of APbPc. Similarly, a decrease of 93.2 J/g was observed in the exothermic enthalpy (Δ*H*) the recorded values were 275.0 and 181.8 J/g for the neat P-ddm resin and P-ddm/APbPc 25 wt.% blend. This indicates that adding APbPc can effectively reduce the curing temperature and curing time of the benzoxazine monomer. This is because the aminoethoxy substituent group on the large APbPc ring is very active, which can catalyze the ring-opening reaction of P-ddm monomer. The hydroxyl groups from the ring-opening polymerization of P-ddm can be used to further catalyze ring-opening polymerization or to react with the aminoethoxy group on the large APbPc ring. The poly(P-ddm) macromolecular chain is connected by four amino bonds on the APbPc ring to form a three-dimensional network structure. The increase of network density of the copolymer increases the mechanical strength. From the above analysis, we can infer the reaction mechanism of P-ddm/APbPc copolymer as Scheme 1.

### 3.3. Curing Kinetics

**Figure 3:**
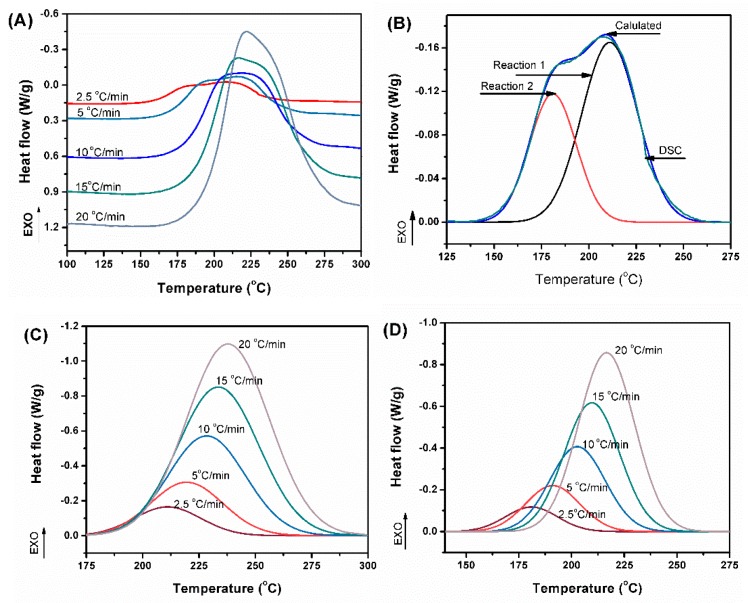
DSC thermograms of P-ddm/APbPc blends at different heating rates (**A**), Lorentz fitting of P-ddm/APbPc blend curve at 2.5 K/min (**B**), reaction 1 curves (**C**), and reaction 2 curves (**D**) at different heating rates.

The curing kinetics of P-ddm/APbPc blends were performed with non-isothermal DSC scans at 2.5, 5, 10, 15, and 20 °C/min heating rates, as shown in Figure 3. The apparent activation energy of the polymerization process was determined by using the various models.

The curing reaction characteristics, such as initial curing temperature, peak curing temperature, and final curing temperature, for the poly(P-ddm/APbPc) copolymer can be obtained from Figure 3A. We can easily observe from Figure 3A that a wide peak was formed in all the curves, which suggests the overlapping of two peaks. Therefore, the curing reaction of copolymer has at least two curing stages, a low temperature amino catalyzed curing reaction and a high temperature simultaneous amino catalytic and autocatalytic curing reaction.

The DSC spectra of the P-ddm/APbPc blend at 2.5 °C/min heating rate was selected to observe the overlapping of the exothermic peaks. The curve was analyzed by using the Lorentz fitting peak separation program in Origin software, and the produced curves are plotted in Figure 3B. The analysis of the DSC spectra confirmed that there were two analytical peaks, reaction 1 and reaction 2, respectively. The resolved data of reactions 1 and 2 at different heating rates are summarized in Figure 3C,D. From the enthalpies of the peaks, we can conclude that two reactions were observed, where reaction 1 has higher proportion in the thermal reaction, relevant data are summarized in Table 2.

**Figure 4:**
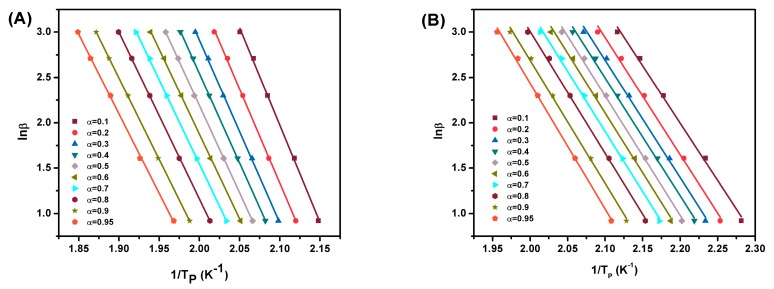
*ln*β versus *1/T_p_* plots for reaction 1 (**A**) and reaction 2 (**B**).

**Figure 5:**
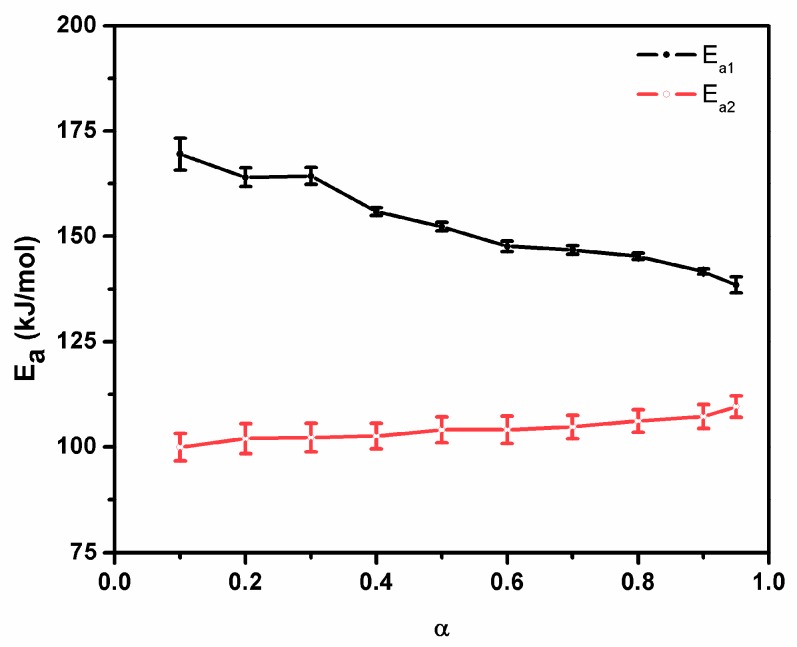
Variation in activation energy values according to the Flynn–Wall–Ozawa (FWO) method for P-ddm/APbPc blends.

According to FWO method and Equation (7), the activation energy (*E_a_*) can be obtained through the slope of the linear fit of *ln*β versus the inverse temperature (*1/T_p_*) plot with various conversion values (α =, 0.1, 0.2, 0.3, 0.4, 0.5, 0.6, 0.7, 0.8, 0.9, 0.95), as shown in Figure 4. The calculated activation energy values as a function of conversion are shown in Figure 5. The average value of the activation energy for reaction 1 (*E_a_*_1_) and reaction 2 (*E_a_*_2_) were 152.57 and 104.28 kJ/mol, respectively.

**Figure 6:**
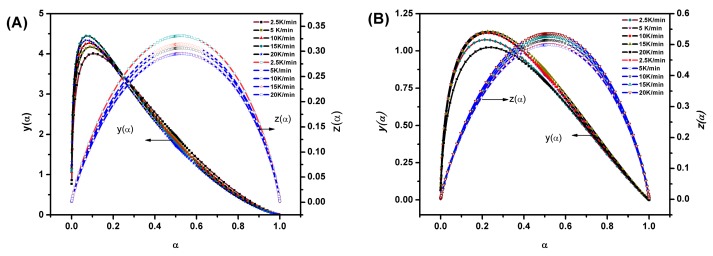
Normalized *y*(*a*), *z*(*a*) versus (*α*) plots for P-ddm/APbPc system, reaction 1 (**A**) and reaction 2 (**B**).

Furthermore, the Málek method was used to determine the suitable curing kinetic model and parameters. According to Equations (9)–(11), the experimental data and the average value of the activation energy, we can determine the data value of *da/dt*, *y*(*α*), and *z*(*α*). Plots of normalized *y*(*a*), and normalized *z*(*a*) versus (*α*) are shown in Figure 6. The conversion rates corresponding to the peaks of *y*(*a*) vs. *a* and *z*(*a*) vs. *a* curves are defined by *a_M_* and *ap*, respectively, and the results are illustrated in Table 3. From the presented data in Table 3, we can easily observe that *α_M_* and $\alpha_{P}^{\infty }$ of the two reactions have the following results: 0 < *α_M_* < $\alpha_{P}^{\infty }$ and $\alpha_{P}^{\infty }$ ≠ 0.632. According to the judging standard of the Málek method, reaction1 and reaction 2 belong to autocatalytic reactions mold. Equation (8) can be used to fit the non-isothermal curing kinetic of P-ddm/APbPc system [51]. By means of logarithmic transformation, Equation (8) can be transformed into Equation (12):

(12) $$\ln\left(\beta \frac{d\alpha }{dT}\right)+\frac{E_{a}}{RT}=\mathrm{lnA}+\mathrm{nln}\left[\alpha^{m/n}\left(1-\alpha \right)\right]$$

where *m/n* can be calculate by *α_M_* = *m*/(*m* + *n*). Moreover, the values of *n* and *lnA* can be obtained from the slope and intercept of the fitted lines of by plotting the variation of [*ln*(β*da/dT*) + *Ea/RT*] versus *ln*[*a^m/n^*(1 − *a*)] for 0.2 ≤ α ≤ 0.90 plot, respectively, results are already summarized in Table 3. From the summarized results we can conclude the curing kinetic model equations for P-ddm/APbPc, Equations (13) and (14) represent the curing kinetic model equations for reaction 1 and reaction 2, respectively.

(13) $$\frac{d\alpha_{1}}{dt}=5.884\times 10^{15}\exp\left(\frac{18351}{T}\right)\alpha_{1}^{0.145}{\left(1-\alpha_{1}\right)}^{1.522}$$

(14) $$\frac{d\alpha_{2}}{dt}=2.459\times 10^{11}\exp\left(\frac{12543}{T}\right)\alpha_{2}^{0.357}{\left(1-\alpha_{2}\right)}^{1.201}$$

(15) $$\frac{d\alpha }{dt}=\frac{\Delta H_{01}}{\Delta H_{0}}\frac{d\alpha_{1}}{dt}+\frac{\Delta H_{02}}{\Delta H_{0}}\frac{d\alpha_{2}}{dt}$$

**Figure 7:**
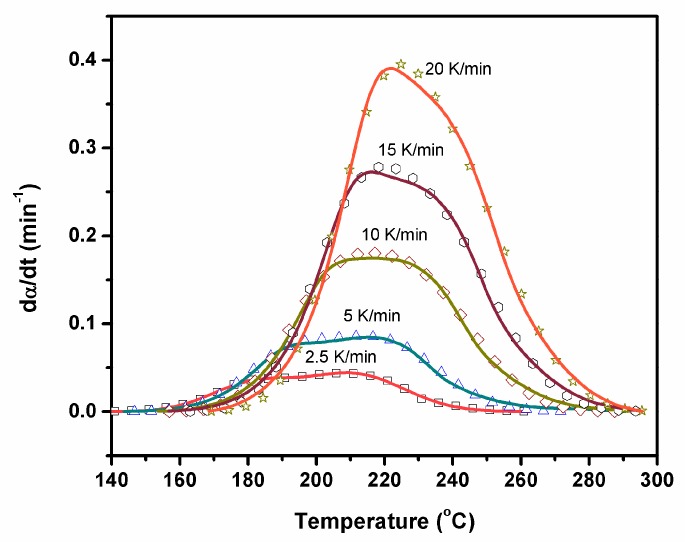
Comparison of experimental values (lines) with autocatalytic model calculated values (symbols).

The overall heat is affiliated with the heat of reactions 1 and reaction 2. Therefore, the overall curing rate, *dα/dt*, is a function of the curing rates of reaction 1 and reaction 2 (dα_1_/dt and dα_2_/dt,), according to Equation (15) [52,54]. In the current study, α = 0.64α_1_ + 0.36α_2_ for P-ddm/APbPc system, which means *dα/dt = 0.64d*α_1_*/dt + 0.36d*α_2_*/dt*. The experimental results were compared with the model predictions, as shown in Figure 7. The calculated results of the model are in good agreement with the experimental results.

### 3.4. Impact Strength of Copolymers

**Figure 8:**
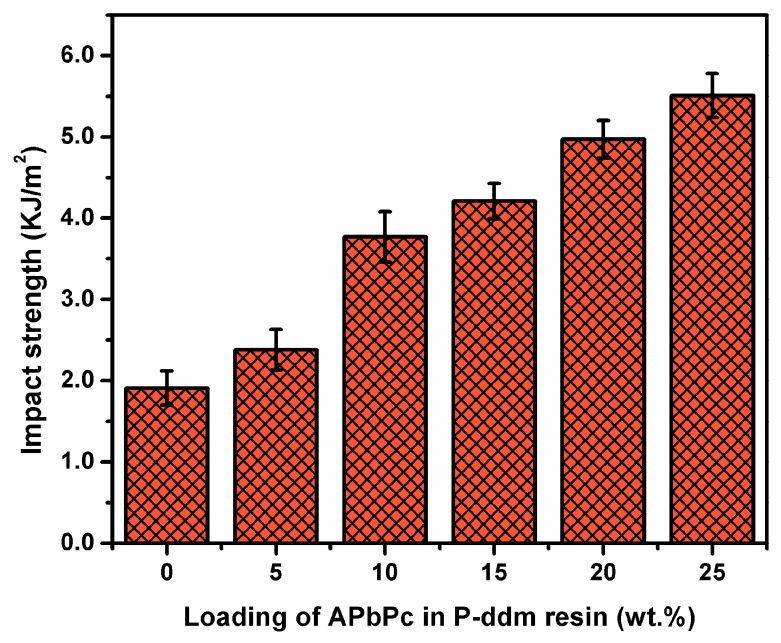
Impact strength of poly(P-ddm/APbPc) on various wt.% loading of APbPc.

The change in the impact strength values as a function of various wt.% of APbPc contents is shown in Figure 8. The highest impact strength value (5.51 ± 0.27 kJ·m^−2^) was recorded for the 25 wt.% APbPc blended copolymer, which is almost 188% higher than recorded value for the neat poly(P-ddm) (1.91 ± 0.21 kJ·m^−2^). These distinctive improvements in the impact strength can be attributed to the flexible aminoethoxy group chain segment of APbPc, which disperses the applied impact load by the segmental movement of aminoethoxy group in the P-ddm/APbPc copolymer. Additionally, a similar cavity formed by a crosslinked matrix structure could absorb some applied energy during impact.

### 3.5. Dynamic Mechanical Analysis

**Figure 9:**
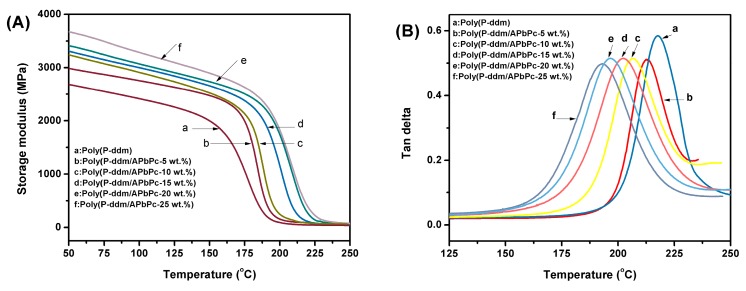
Storage modulus (**A**) tan delta (**B**) of P-ddm/APbPc copolymer at different mass fraction of APbPc.

Dynamic mechanical analysis of P-ddm/APbPc copolymers was carried out as a temperature function to ascertain the thermomechanical behavior of the copolymer and analyze changes in dynamic modulus and glass transition temperature (*T_g_*) with respect to increasing wt.% of APbPc in copolymer. The variations of storage modulus (*G′*) and damping factor (tan δ) against temperature are illustrated in Figure 9. The stiffness (*G′* at 50 °C), *T_g_* (tan δ peak temperature), and crosslinking density calculated by the rubbery equation are summarized in Table 4.

*G′* is an indicator of matrix stiffness under shear deformation state. We can easily observe from Figure 9A, that *G′* was gradually increased as the mass fraction of APbPc was raised in the copolymer. The *G′* value of poly(P-ddm) at 50 °C was recorded as 2675.9 MPa, and a rise of 1003 MPa was observed as for 25 wt.% containing APbPc copolymer, the recorded value was 3678.9 MPa. Here APbPc reacts with P-ddm, and forms a three-dimensional crosslinking structure. Moreover, APbPc is a conjugated macrocyclic compound with four substituted in the periphery with aminoethoxy groups. APbPc blending improves the crosslink densities of the copolymers, which enhances the capabilities of the storage energy as compared to neat resins [58]. Similarly, the crosslinking density was increased as the mass share of APbPc was raised from 0 to 25 wt.% as shown in Table 4; the recorded values were 1617.8 and 6783.2 mol/m^3^.

The single tan δ transition peak was observed in all the studied copolymer compositions within 100 to 250 °C temperature range as shown in Figure 9B. The *T_g_* of copolymers with different APbPc proportions can be obtained from tan δ transition peak. The addition of APbPc in the copolymer matrix leads to the decrease of *T_g_*, due to the flexible chain in APbPc, and which can have the chain segmental movement at relatively low temperature. The *T_g_* value of pure poly(P-ddm) was recorded at 217.6 °C, and *T_g_* value of 25 wt.% APbPc containing copolymer was recorded 24.6 °C lower (193.0 °C).

### 3.6. Thermogravimetric Analysis

**Figure 10:**
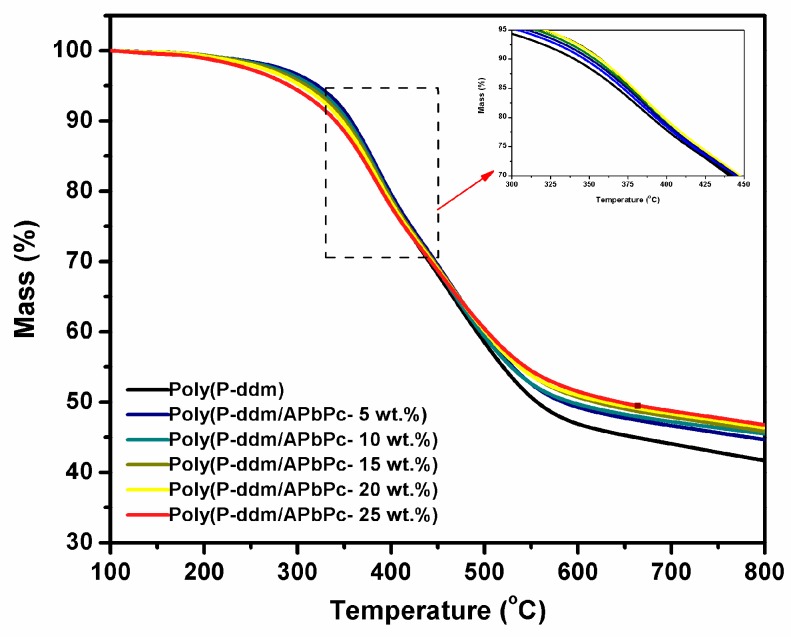
TGA curves of P-ddm/APbPc copolymers on different wt.% blending of APbPc.

The thermal decomposition of the copolymers was employed by TGA under nitrogen atmosphere from room temperature to 800 °C at 10 °C/min heating rate. TGA curves are presented in Figure 10, and Table 5 presents the detailed thermal decomposition temperature values in terms of 5 wt.% (*T_5%_*) and 10 wt.% (*T_10%_*) losses along with the percentage residual weight (*Y_c_*) taken at 800 °C.

The thermal degradation curves of poly(P-ddm/APbPc) were sigmoidal shaped. From the summarized data, we can observe that the initial thermal decomposition temperatures (*T_5%_* and *T_10%_*) of poly(P-ddm/APbPc) were slightly lower than the recorded values of neat poly(P-ddm). The *T_5%_* and *T_10%_* values of pristine poly(P-ddm) were recorded as 320.6 and 357.8 °C, respectively, the value reduced to 290.7 and 340.2 °C for 25 wt.% loaded APbPc, respectively. The *T_5%_* and *T_10%_* of copolymers were slightly lower because of the poor relative stability of the aminoethoxy group of APbPc. However, the situation reversed in between 357 and 425 °C, the thermal stability of poly(P-ddm/APbPc) increased as the share of APbPc was raised. The ultimate impact was confirmed by analyzing the *Y_c_* values, the *Y_c_* value was increased from 41.6% to 46.8%, as the share of APbPc was increased from 0 to 25 wt.%. These improvements in the thermal stability parameters of copolymers can be dedicated to the amino groups containing APbPc monomer, which have good thermal resistance at moderate temperature, and formed very high crosslinked macromolecule structure. These results strongly correlate with the earlier discussed DMA data and the impact strength results. This confirms that the produced copolymers possess higher thermal stabilities as compared to the pristine poly(P-ddm).

## 4. Conclusions

In the current study, a high-performance poly(P-ddm/APbPc) copolymer was prepared. The blending of APbPc lowered the curing temperature of the P-ddm matrix due to the reactive –NH_2_ groups. The curing behavior of the P-ddm/APbPc blends before and after curing was analyzed by FTIR and DSC tests. According to the molecular structure and reaction mechanism, the three-dimensional network structure and toughening mechanism of the cured system were predicted. The curing kinetics was studied by a non-isothermal method, and the kinetic equations between curing degree and curing temperature and time were obtained. The DMA and impact strength tests proved that the best properties were found for the poly(P-ddm/APbPc-25 wt.%) specimen. An increase of 1003 MPa in the copolymer stiffness and 24.6 °C decline in *T_g_* temperature was observed. The TGA curves under nitrogen atmosphere showed that the copolymers also exhibit excellent thermal properties. This study confirmed that the high-performance benzoxazine thermosetting resin performance can be improved by blending the APbPc monomer.

## Figures and Tables

**Table 1:** DSC data for P-ddm/APbPc blends at different wt.% of APbPc.

APbPc (wt.%)	*T_o_* (°C)	*T_p_* (°C)	*T_f_* (°C)	Δ*H* (J/g)
0	227.2	241.0	267.3	275.0
5	211.2	233.2	267.6	272.1
10	204.5	229.3	267.2	259.4
15	195.8	226.3	267.4	224.9
20	192.1	224.7	267.5	213.4
25	188.6	222.5	268.0	202.8

**Table 2:** The proportions of two reactions in P-ddm/APbPc blends at different heating rates.

Heating Rate (°C/min)	Δ*H*_0_ (J/g)	Δ*H*_01_ (J/g)	Δ*H*_02_ (J/g)	Δ*H*_01_/Δ*H*_0_	Δ*H*_02_/Δ*H*_0_
**2.5**	235.42	147.44	87.98	0.63	0.37
**5**	236.66	150.04	86.62	0.63	0.37
**10**	237.83	153.90	83.93	0.65	0.35
**15**	238.27	155.69	82.58	0.65	0.35
**20**	238.72	152.83	85.89	0.64	0.36
**Mean**	237.38	151.98	85.40	0.64	0.36

**Table 3:** The kinetic parameters calculated for the P-ddm/APbPc curing system.

β (K/min)	α_M1_	$\alpha_{P1}^{\infty }$	*m*_1_	*n*_1_	*lnA*_1_	α_M2_	$\alpha_{P2}^{\infty }$	*m*_2_	*n*_2_	*lnA*_2_
2.5	0.105	0.531	0.170	1.453	36.323	0.223	0.525	0.350	1.220	26.209
5	0.092	0.538	0.152	1.504	36.318	0.219	0.533	0.344	1.227	26.262
10	0.086	0.524	0.143	1.524	36.312	0.231	0.521	0.361	1.201	26.250
15	0.077	0.517	0.132	1.571	36.323	0.233	0.541	0.359	1.181	26.256
20	0.074	0.528	0.125	1.560	36.282	0.240	0.541	0.371	1.175	26.161
Mean			0.145	1.522	36.311			0.357	1.201	26.228

**Table 4:** Summary of dynamic mechanical analysis (DMA) data and crosslinking density values of P-ddm/APbPc copolymer at different mass fractions.

APbPc (wt.%)	*G′* (MPa @ 50 °C)	*T_g_* (°C)	ρ (mol/m^3^)
0	2675.9	217.6	1617.8
5	2991.0	212.8	2894.9
10	3241.2	206.6	4335.9
15	3303.7	202.4	4935.6
20	3413.1	196.6	5396.7
25	3678.9	193.0	6783.2

**Table 5:** TGA data for P-ddm/APbPc copolymers on different wt.% blending of APbPc.

APbPc (wt.%)	*T_5%_* (°C)	*T_10%_* (°C)	*Y_c_* (%, at 800 °C)
0	320.6	357.8	41.6
5	319.6	356.8	44.6
10	315.5	355.7	45.5
15	310.3	350.6	45.8
20	303.1	346.4	46.3
25	290.7	340.2	46.8

## Supplementary Materials

The following are available online at https://www.mdpi.com/2073-4360/11/11/1855/s1.
