# Erratum: Amino-Functionalized Lead Phthalocyanine-Modified Benzoxazine Resin: Curing Kinetics, Thermal, and Mechanical Properties. *Polymers* 2019, *11*, 1855

**DOI:** 10.3390/polym12010002

**Published:** 2019-12-18

**Authors:** Li-wu Zu, Bao-chang Gao, Zhong-cheng Pan, Jun Wang, Abdul Qadeer Dayo, Wen-bin Liu

**Affiliations:** 1 Institute of Composite Materials, College of Materials Science and Chemical Engineering, Harbin Engineering University, Harbin 150001, China; zlwsm@163.com (L.-w.Z.); chemgbc@hrbeu.edu.cn (B.-c.G.); 2 College of Materials Science and Engineering, Heilongjiang Province Key Laboratory of Polymeric Composition, Qiqihar University, Qiqihar 161006, China; 3 Department of Chemical Engineering, Engineering and Management Sciences, Balochistan University of Information Technology, Quetta 87300, Pakistan

The authors wish to make a change to the published paper [1]. In the original manuscript, there is a mistake on the structure of P-ddm. The corrected Scheme 1 is presented below. 

The authors apologize for any inconvenience caused and the change does not affect the scientific results. The manuscript will be updated and the original will remain online on the article webpage at https://www.mdpi.com/2073-4360/11/11/1855.

## Figures and Tables

**Scheme 1:**
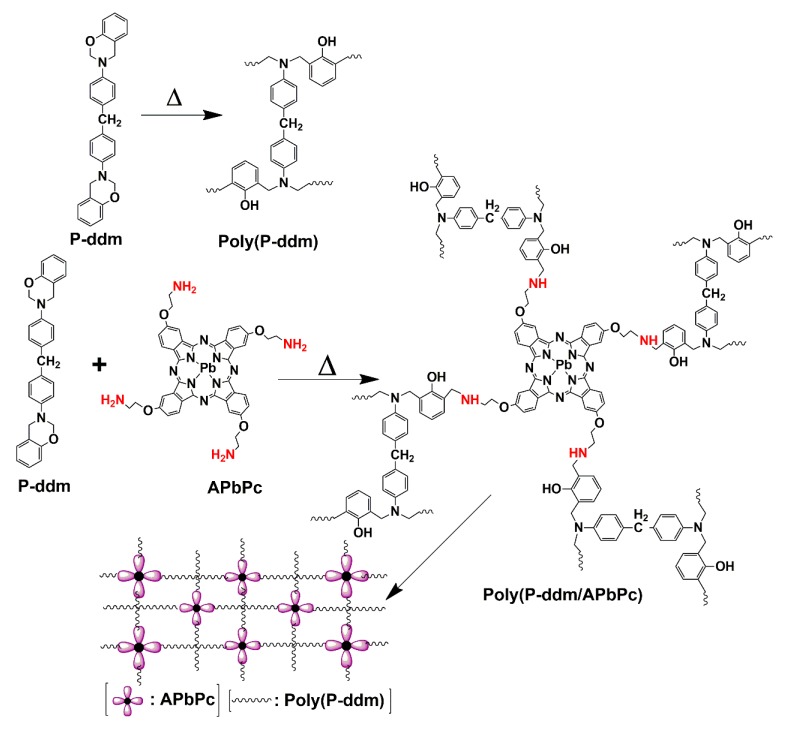
Proposed crosslinked network structure for P-ddm/APbPc copolymer.
