# Supplementary material for: Amino-Functionalized Lead Phthalocyanine-Modified Benzoxazine Resin: Curing Kinetics, Thermal, and Mechanical Properties
Source: Polymers (Basel). 2019 Nov 11;11(11):1855. doi: 10.3390/polym11111855 (PMC6918423; doi:10.3390/polym11111855)
Supplement: Supplementary file 1 [file polymers-11-01855-s001.pdf]

Amino-Functionalized Lead Phthalocyanine-Modified Benzoxazine Resin: Curing Kinetics, Thermal, and Mechanical Properties

Li-wu Zu [1,2], Bao-chang Gao [1], Zhong-cheng Pan [1,*], Jun Wang [1,*], Abdul Qadeer Dayo [1,3,*] and Wen-bin Liu [1,*]

1 Institute of Composite Materials, College of Materials Science and Chemical Engineering, Harbin Engineering University, Harbin 150001, China

2 College of Materials Science and Engineering, heilongjiang province Key Laboratory of Polymeric Composition, Qiqihar University, Qiqihar 161006, China

3 Department of Chemical Engineering, Engineering and Management Sciences, Balochistan University of Information Technology, Quetta 87300, Pakistan

* Correspondence: 421336390@herbeu.edu.cn (Z.P.); wj6267@hrbeu.edu.cn (J.W.); abdul.qadeer@buitms.edu.pk (A.Q.D.); liuwenbin@hrbeu.edu.cn (W.L.)

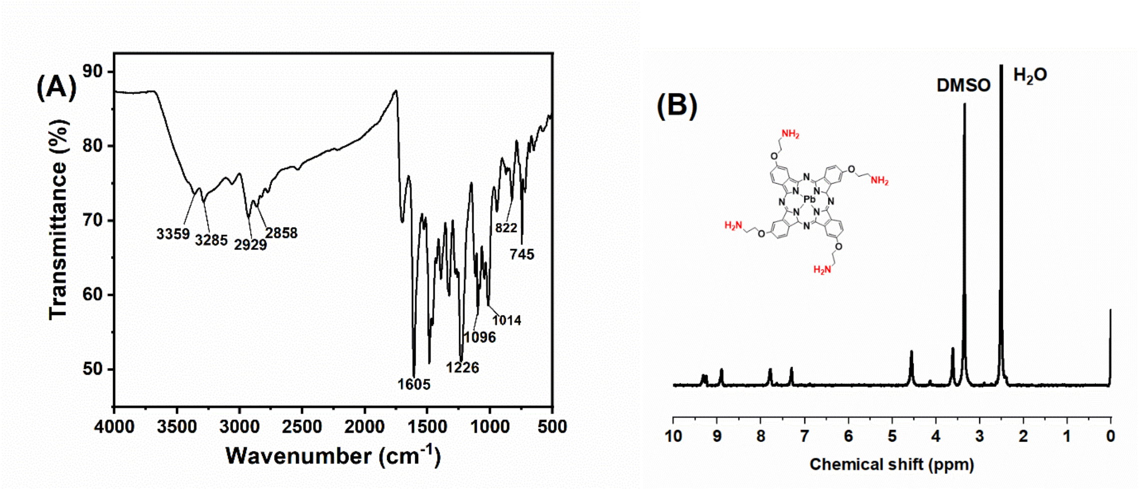

Figure S1. FTIR and ^{1}HNMR spectra of the APbPc monomer.

^{1}H NMR (600MHz, DMSO d_6) ppm δ: 9.30 (d, 4H), 8.89 (s, 4H), 7.79 (s. 4H), 7.29 (s, 8H) 4.55 (s, 8H), 3.61 (s, 8H). FT-IR [(KBr) υ_{max}/cm^{-1}]: 3359, 3285, 2929, 2858, 1605, 1226, 1096, 1014, 822, 745, [44, 45, 56].
